# Mitochondrial DNA Damage and Dysfunction, and Oxidative Stress Are Associated with Endoplasmic Reticulum Stress, Protein Degradation and Apoptosis in High Fat Diet-Induced Insulin Resistance Mice

**DOI:** 10.1371/journal.pone.0054059

**Published:** 2013-01-16

**Authors:** Larysa V. Yuzefovych, Sergiy I. Musiyenko, Glenn L. Wilson, Lyudmila I. Rachek

**Affiliations:** Department of Cell Biology and Neuroscience, University of South Alabama, Mobile, Alabama, United States of America; University of Medicine and Dentistry of New Jersey, United States of America

## Abstract

**Background:**

Recent studies showed a link between a high fat diet (HFD)-induced obesity and lipid accumulation in non-adipose tissues, such as skeletal muscle and liver, and insulin resistance (IR). Although the mechanisms responsible for IR in those tissues are different, oxidative stress and mitochondrial dysfunction have been implicated in the disease process. We tested the hypothesis that HFD induced mitochondrial DNA (mtDNA) damage and that this damage is associated with mitochondrial dysfunction, oxidative stress, and induction of markers of endoplasmic reticulum (ER) stress, protein degradation and apoptosis in skeletal muscle and liver in a mouse model of obesity-induced IR.

**Methodology/Principal Findings:**

C57BL/6J male mice were fed either a HFD (60% fat) or normal chow (NC) (10% fat) for 16 weeks. We found that HFD-induced IR correlated with increased mtDNA damage, mitochondrial dysfunction and markers of oxidative stress in skeletal muscle and liver. Also, a HFD causes a change in the expression level of DNA repair enzymes in both nuclei and mitochondria in skeletal muscle and liver. Furthermore, a HFD leads to activation of ER stress, protein degradation and apoptosis in skeletal muscle and liver, and significantly reduced the content of two major proteins involved in insulin signaling, Akt and IRS-1 in skeletal muscle, and Akt in liver. Basal p-Akt level was not significantly influenced by HFD feeding in skeletal muscle and liver.

**Conclusions/Significance:**

This study provides new evidence that HFD-induced mtDNA damage correlates with mitochondrial dysfunction and increased oxidative stress in skeletal muscle and liver, which is associated with the induction of markers of ER stress, protein degradation and apoptosis.

## Introduction

Consumption of a Western diet, which is high in saturated fat (high fat diet, (HFD)) is associated with obesity, metabolic syndrome, insulin resistance (IR), type 2 diabetes, and cardiovascular diseases. Studies in humans and animals have shown a link between lipid accumulation in non-adipose tissue, such as skeletal muscle and liver, and IR [1]–[5]. Although the mechanisms responsible for IR in these tissues are different [5], mitochondrial dysfunction and oxidative stress are considered major risk factors for the pathogenesis of IR [6]–[8]. A HFD-induced increase in oxidative stress in skeletal muscle has been proposed as a unifying mechanism promoting mitochondrial dysfunction, lipid accumulation, and IR [6]. In addition, a more recent study has demonstrated that mitochondrial superoxide production is a unifying element in IR, including HFD-induced IR in skeletal muscle [9]. Also, related to hepatic IR, a recent study has demonstrated that mitochondrial dysfunction precedes IR and hepatic steatosis in an obese rat model [8]. Furthermore, it has been shown that the saturated free fatty acid (FFA) palmitate induced IR in hepatocytes *in vitro* through increased mitochondrial oxidative stress [10].

For the last two decades, extensive studies on obese humans with metabolic syndrome and type 2 diabetes, as well as on animals models of obesity and IR have been performed. These include studies on mitochondria, endoplasmic reticulum (ER) stress and protein degradation, but the molecular events triggering the pathways leading to the development of IR are yet to be clarified. The topic of whether diet induced obesity (DIO) caused mitochondrial dysfunction in skeletal muscle is still highly controversial, with some reports linking HFD-induced IR to mitochondrial dysfunction [6]–[7], whereas other have reported that a HFD caused IR despite an increase in muscle mitochondrial density and oxidative capacity [11]–[12]. Since IR is associated with numerous modern health problems, including type 2 diabetes and cardiovascular disease, it is an urgent priority to establish the molecular targets and upstream events that mediate the development of IR. Among the potential targets is mitochondrial DNA (mtDNA), since mtDNA is highly specialized and encodes for proteins essential for energy metabolism. In addition, in a recent study, we discovered that mtDNA integrity plays a crucial role in both mitochondrial dysfunction and IR by showing that palmitate-induced damage to mtDNA heightens mitochondrial reactive oxygen species (ROS) production and mitochondrial dysfunction, thus impairing insulin signaling [13]. Therefore, we believe that it also is critical to evaluate whether IR correlates with compromised integrity of mtDNA *in vivo.* Considering that no previous reports have been shown which link mtDNA damage with IR, mitochondrial dysfunction, and oxidative stress in the two major sites of IR development, skeletal muscle and liver, in an obesity-induced model of IR, the aim of this study was to determine whether a HFD induced 1) mtDNA damage and if so, whether this damage associates with 2) mitochondrial dysfunction, 3) oxidative stress, 4) change in the expression of the DNA repair enzymes; 5) induction of endoplasmic reticulum (ER) stress, and 6) protein degradation and apoptosis in two peripheral insulin responsive tissues, skeletal muscle and liver in a mouse model of obesity and IR. This is the first study which shows that there is a positive correlation between mtDNA damage, mitochondrial dysfunction, oxidative stress and activation of markers of both ER stress and degradation of proteins, apoptosis and development of IR in skeletal muscle and liver.

## Materials and Methods

### Animals and Measurement of Metabolic Parameters

C57BL/6J male mice were fed either a HFD (60% fat (of which 90 % was lard and 10% soybean oil by calories), 20% protein, and 20% carbohydrate by calories, 5.24 kcal/g metabolizable energy; diet no. D12492) or normal chow (NC) (10% fat, 20% protein and 70 % carbohydrate, 3.8 kcal/g metabolizable energy; diet no D12450B) (Research Diets, New Brunswick, NJ) for 16 weeks, starting at 6 weeks of age. HFD/NC fed mice were from Jackson Laboratory (Sacramento, CA). All procedures used in this study were approved by the Institutional Animal Care and Use Committees of The Jackson Laboratory (Sacramento, CA, approval number JW10011) and University of South Alabama (approval number 07025) and fully complied with the guidelines from the National Institute of Health. Blood was withdrawn in a fasted state from the orbital sinus of anesthetized animals with heparinized microcapillary tubes and serum was isolated using a BD Microtainer (Franklin Lakes, NJ) according to the manufacturer’s instructions. Metabolic parameters were measured as described elsewhere [7]. Insulin levels were measured using an Ultrasensitive Insulin Elisa Kit from Crystal Chem, Inc. (Downers Grove, IL). FFA level was determined by standard colorimetric method using a kit from Wako Chemicals (Richmond, VA) and triglycerides by a triglycerides/Glycerol blanked kit from Roche Diagnostics (Indianapolis, IN). For oral glucose tolerance test (OGTT), after a 12 h fast, a 20% glucose solution, (2 g/kg) was administered orally to mice. For insulin tolerance test (ITT) after 8 h food deprivation, insulin (0.1 u/kg) was injected i.p. For both analyses, blood samples were taken from the tail at the indicated times and blood glucose concentrations were measured using a Glucose HK Gen.3 kit from Roche Diagnostics (Indianapolis, IN). At the end of the protocols, mice were sacrificed by cervical dislocation, and liver and mixed gastrocnemius muscle were rapidly excised and frozen in liquid nitrogen.

### Oxidative Stress, Protein Carbonylation and ATP Measurement

Oxidative stress in both skeletal muscle and liver was examined using a glutathione assay kit from Cayman Chemical (Ann Arbor, MI) according to the manufacturer’s instructions. Oxidative protein carbonylation assays in both skeletal muscle and liver were performed following Western blot by using an OxyBlot Protein Detection Kit from Millipore (Billerica, MA) according to the manufacturer’s instructions. The carbonyl groups in protein side chains were derivatized to DNP-hydrazone by reaction with DNPH following the manufacturer’s instructions. After the derivatization of the protein sample, 1-dimensional electrophoresis was carried out on a 10% SDS-PAGE gel. Proteins were transferred to PVDF membranes. After incubation with anti-DNP antibody, the blot was developed using a chemiluminescence detection system. ATP levels were analyzed in liver and skeletal muscle extracts as described previously [14]. ATP concentrations were determined using the luciferase-based ATP-assay from Roche (Mannheim, Germany), and values were normalized to mtDNA content.

### Mitochondrial DNA Damage and Copy Number Analysis

Liver and gastrocnemius skeletal muscle from both groups of animals were homogenized in liquid nitrogen and incubated in lysis buffer [10 mM TRIS-HCI (pH 8.0), 20 mM EDTA (pH 8.0), 100 mM NaCl, 0.75% SDS and 0.3 mg/ml proteinase K] overnight at 37°C. To evaluate mtDNA damage, quantitative alkaline Southern blot analysis was performed to evaluate changes in the density of mtDNA lesions as described previously with minor modifications [13]–[15]. Briefly, after isolation of total DNA, DNA was digested with EcoRI, precisely quantified and quantitative alkaline Southern blot was performed using a mouse mtDNA specific probe (cytochrome c oxidase, I subunit). To evaluate nuclear DNA (nDNA) damage, quantitative alkaline Southern blot analysis was performed using a mouse nDNA specific probe (IgE). To ensure that HFD-induced mtDNA damage did not reflect changes due to an alteration in mtDNA content, a slot blot analysis [16] was performed using the same samples of DNA. We used 0.5–2 µg of total DNA prepared from muscle samples. The DNA was denatured by 0.3 M NaOH, linked to a nylon membrane in a GS Gene Linker (Bio-Rad, Hercules, CA) and probed with a mitochondrial (cytochrome c oxidase, I subunit) or nuclear (IgE) probe. Hybridization images were scanned and band intensities were determined, which allowed a direct comparison between the amount of mtDNA and nDNA present at each sample. For mtDNA copy number, relative values from band intensities (mtDNA/nDNA) were calculated by comparing each sample with average of NC fed mice. Results were normalized by the mean value of the NC condition set to 1 unit and presented as mtDNA/nDNA ratio. For mtDNA or nDNA damage, the resultant band intensities obtained after quantitative alkaline Southern blot analysis were normalized by the mean value of the NC condition set to 1 unit and presented as arbitrary units (A.U.). Additionally, the results for mtDNA damage obtained after quantitative alkaline Southern blot analysis were normalized for mtDNA copy number.

### Subcellular Fractionation, Protein Isolation, Immunoprecipitation and Western Blot Analysis

For isolation of protein extracts for total cellular fractions, frozen gastrocnemius skeletal muscle and liver from both groups of animals were homogenized in liquid nitrogen and incubated in Cell Lysis Buffer (Cell Signaling, Beverly, MA) supplemented with 0.1 mg PMSF and a 1/100 dilution of protease and phosphatase inhibitor cocktails (Sigma St. Louis, MO). The extracts additionally were homogenized. Samples were centrifuged for 10 min at 14,000 g and the supernatants were used for Western blots. Subcellular fractions were isolated as described in our previous publications for cell culture, with minor modifications [13]–[15]. For isolation of nuclear, mitochondrial and cytosolic protein fractions, frozen gastrocnemius skeletal muscle and liver from both groups of animals were homogenized in liquid nitrogen and resuspended in isolation buffer [0.25 M Sucrose, 1 mM EDTA (pH 8.0), 10 mM TRIS-HCI (pH 7.4)] supplemented with 0.1 mg PMSF and a 1/100 dilution of protease and phosphatase inhibitor cocktails (Sigma St. Louis, MO). Protein concentrations were determined using the Bio-Rad protein dye micro-assay (Bio-Rad, Hercules,CA). Acrylamide gel electrophoresis and transfer of separated proteins to PVDF-membranes were performed as previously described [13]–[15]. Blocking and antibody immunoblotting were performed in 5% nonfat dry milk and Tris-buffered saline with 0.1% Tween 20 (TBS-T). TBS-T and TBS were used for washing. The antibodies used were to actin (Sigma); caspase-3, cleaved caspase-3, GAPDH, phospho-Akt (Ser 473), total Akt, phospho-SAP/JNK (T183/Y185), total SAP/JNK, IRS-1 (Cell Signaling; Beverly, MA); lamin A, PGC-1α, TFAM and CHOP (Santa Cruz Biotechnology, Santa Cruz, CA), OGG1 from Novus Biologicals (Littleton, Colorado), APE1 (Abcam, Cambridge, MA), cytochrome c (PharMingen; San Diego, CA). Where indicated, the resultant band images were scanned and analyzed using Fujifilm Image Gauge Version 2.2 software. For all densitometry analyses of Western blots, a value of 1 was arbitrary assigned to NC conditions to which HFD were reported and expressed as fold basal as described previously [17].

### Statistical Analysis

Data are expressed as means ± SE. Differences between two groups were assessed using unpaired, two-tailed Student’s t-test. Statistical significance was determined at the 0.05 level.

## Results

### Metabolic Characteristics after a HFD

The metabolic characterictics are summarized in Table S1. Sixteen weeks of HFD feeding induced conditions which closely resemble metabolic syndrome: HFD-fed mice gain significantly more weight, have higher serum glucose, insulin, triglycerides and FFAs levels. In addition, OGTT and ITT tests revealed that HFD-fed animals have impaired glucose tolerance (Fig. S1A) and were insulin resistant (Fig. S1B).

### HFD Induced Oxidative Stress and Decreased ATP Levels in Both Skeletal Muscle and Liver

**Figure 1:**
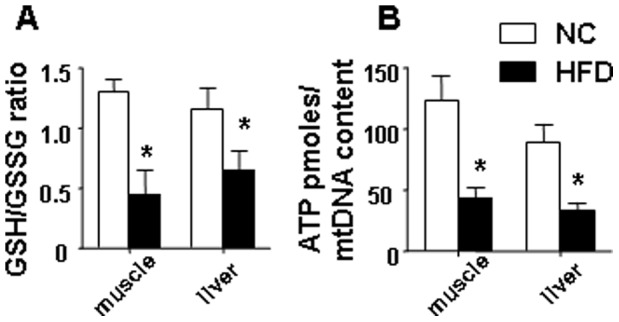
HFD induced oxidative stress, and decreased ATP levels in skeletal muscle and liver. (A) the GSH/GSSG ratio was significantly decreased in both gastrocnemius muscle and liver samples isolated from mice fed a HFD. (B) a HFD reduced the ATP levels in both skeletal muscle and liver. ATP concentrations were determined using the luciferase-based ATP-assay, values were normalized to mtDNA content. The average results ± SE are shown. (*p<0.05 vs corresponding NC, n = 7–9 mice per group).

To evaluate whether long term HFD feeding leads to increased oxidative stress in both skeletal muscle and liver, a glutathione assay kit was utilized. The ratio of glutathione (GSH) and glutathione disulfide (GSSG) is a widely-used indicator of oxidative stress and cellular redox environment in tissues. A significant decrease in the GSH/GSSG ratio indicates elevated oxidative stress in the gastrocnemius muscle and liver from HFD fed animals (Fig. 1A). Additionally, we found that a HFD significantly reduced ATP levels in both the gastrocnemius muscle and liver (Fig. 1B). ATP is expressed relative to the mtDNA content of the samples to reflect how much ATP was generated by mitochondria.

### HFD Increased Mitochondrial DNA Damage in Both Skeletal Muscle and Liver

**Figure 2:**
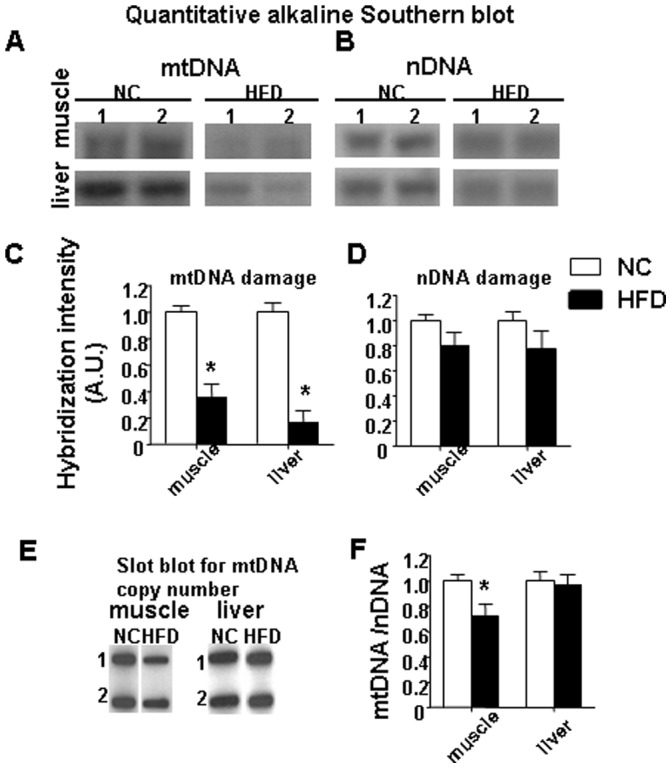
HFD damaged mtDNA to a greater extent than nDNA in both skeletal muscle and liver. Representative autoradiograms from Southern blot analyses of (A) mtDNA and (B) nDNA are shown for skeletal muscle and liver from two NC and two HFD mice. (C) displays the graphs for mtDNA damage. (D) the graphs for nDNA damage are shown. (E) are representative autoradiograms from slot-blot analyses performed on the DNA from skeletal muscle and liver isolated from two NC and two HFD mice. (F) mtDNA copy number normalized to nDNA copy number in the gastrocnemius muscle and liver. Data represent the means ± SE. (*p<0.05 vs corresponding NC, n = 7–9 mice per group).

Next, we evaluated the effect of HFD diet on mtDNA and nDNA damage and found that HFD damaged mtDNA to a greater extent than nDNA in both skeletal muscle and liver (Fig. 2A–D). To ensure that HFD-induced mtDNA damage did not reflect changes due to alteration of mtDNA content, we performed slot blot analysis using the same samples of DNA. We found that a HFD induced a significant decrease in mtDNA copy number in gastrocnemius muscle (Fig. 2E and F **)**. Interestingly, we did not find any difference in mtDNA copy number in liver samples isolated from HFD or NC mice.

### HFD Induced Phosphorylation/activation of JNK and Increased Protein Carbonylation in Both Skeletal Muscle and Liver

**Figure 3:**
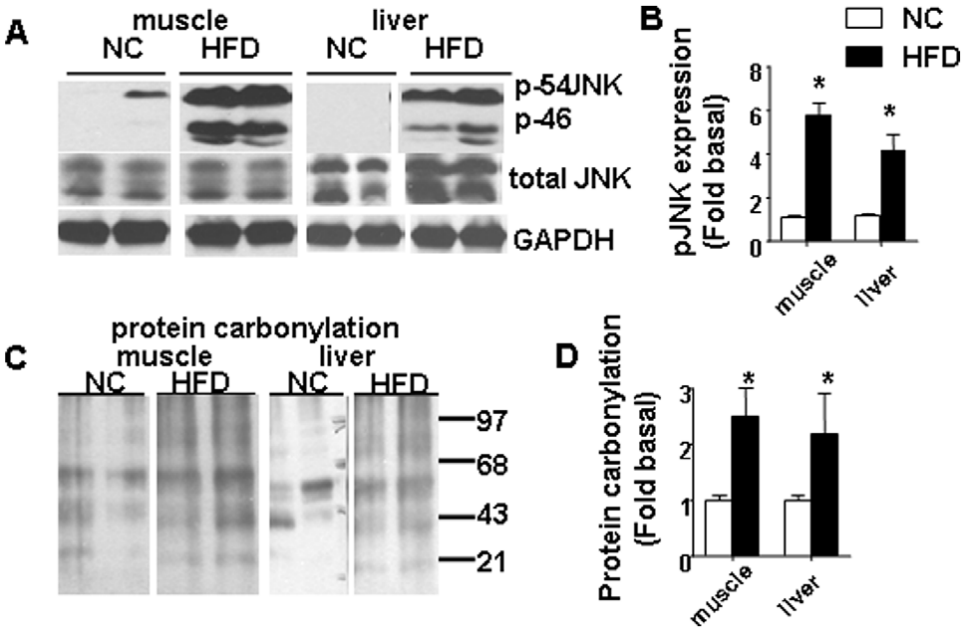
A HFD induced activation of JNK and increased protein carbonylation in skeletal muscle and liver. (A) a representative of immunoblots of phosphorylation (Thr183/Tyr185)/total JNK in gastrocnemius muscle and liver are shown. (B) densitometry data for pJNK are shown. (C) oxidative protein carbonylation in gastrocnemius muscle and liver extracts. (D) densitometry data for protein carbonylation are shown. Data represent the means ± SE. ((* p<0.05 vs corresponding NC, n = 6–9 mice per group).

Since activation of JNK was shown in the conditions of both increased oxidative and ER stress, we next evaluated phosphorylation/activation status for JNK in both skeletal muscle and liver (Fig. 3A). Data were normalized to the density of bands of NC animals as described in “Materials and Methods” (Fig. 3B). We showed that a HFD activated phosphorylation of JNK kinase in both skeletal muscle and liver (Fig. 3A and B). In agreement with a decreased GSH/GSSG ratio and activation of JNK, additional markers of increased oxidative stress, protein carbonylation levels were correspondingly increased in both skeletal muscle and liver samples isolated from the HFD group (Fig. 3C and D).

### Effect of HFD on Mitochondrial Protein Content and DNA Repair Proteins in Skeletal Muscle and Liver

**Figure 4:**
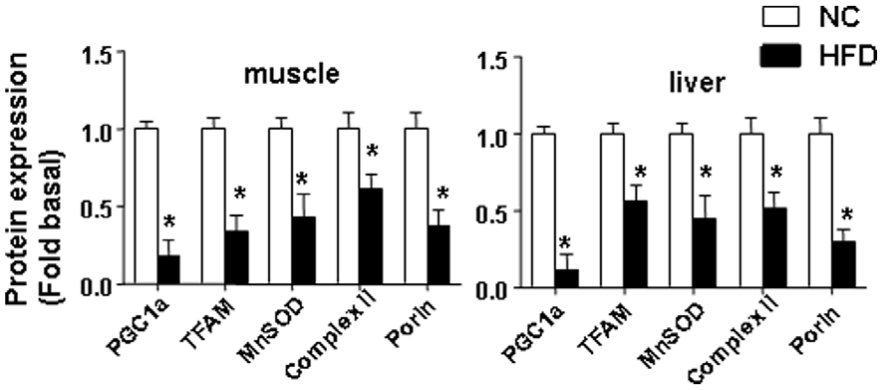
HFD reduced protein content of transcription factors involved in mitochondrial biogenesis, mitochondrial antioxidants and mitochondrial markers. The average results ± SE are shown. (*p<0.05 vs corresponding NC, n = 6–9 mice per group).

**Figure 5:**
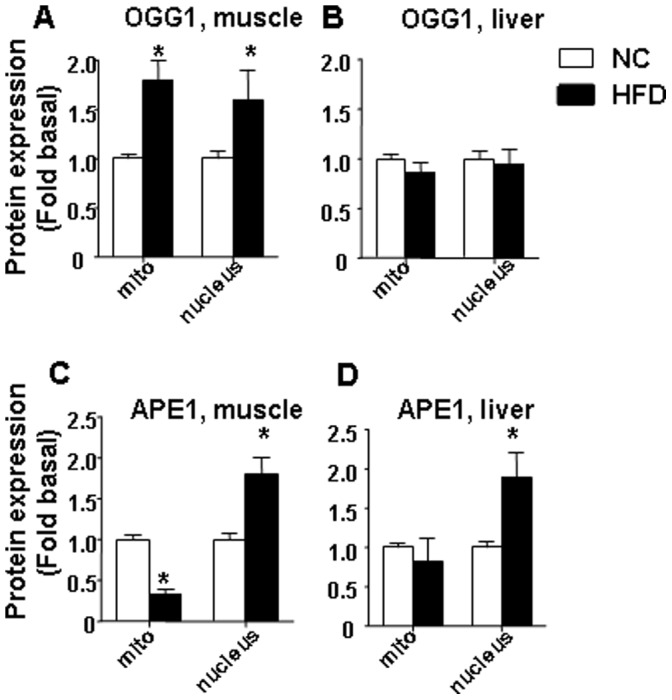
Effect of HFD on proteins involved in BER. Effect of HFD: on nuclear or mitochondrial expression of OGG1 in (A) skeletal muscle and (B) liver; on nuclear or mitochondrial expression of APE1 in (C) skeletal muscle and (D) liver. The average results ± SE are shown. (*p<0.05 vs corresponding NC, n = 6–9 mice per group).

We next examined protein content for mitochondrial proteins and proteins involved in base excision (BER) DNA repair in both nuclear and mitochondrial fractions of samples isolated from HFD or NC fed animals. Consistent with the increased mtDNA damage and reduction of ATP, we found that a HFD reduced mitochondrial protein content, including proteins implicated in mtDNA replication and biogenesis (mitochondrial transcription factor, TFAM) and mitochondrial oxidant defense (Manganese Superoxide Dismutase, MnSOD) in both skeletal muscle and liver (Fig. 4). Levels of the mitochondrial marker protein porin were substantially reduced in both skeletal muscle and liver from HFD fed mice (Fig. 4). Also, consistent with previous reports [7]–[18], a HFD dramatically reduced expression of peroxisome proliferator activator receptor-γ coactivator 1α (PGC-1α) a major protein implicated in mitochondrial biogenesis. Regarding DNA repair enzymes, we compared levels of two major proteins of BER, OGG1 (8-oxoguanine DNA glycosylase/AP lyase) and APE1 (Apurinic/apyrymidinic Endonuclease 1) in skeletal muscle and liver samples isolated from HFD or NC fed animals (Fig. 5). Furthermore, we compared levels of both OGG1 and APE in both nuclear and mitochondrial fractions. First, nuclear, mitochondrial and cytosolic fractions were isolated from skeletal muscle and liver of NC/HFD fed mice and analyzed by Western blot to confirm the purity of fractions (Fig. S2). Lamin A was used as a marker for nuclear proteins, subunit IV of mitochondrial complex IV (Cox IV, Sub. IV) was used as marker for mitochondrial proteins and actin was used as a marker for cytosolic proteins. No detected nuclear contamination was presence in the mitochondria, and no mitochondrial contamination was present in nuclear fractions (Fig. S2). Interestingly, we found that HFD significantly increased protein content for both mitochondrial and nuclear OGG1 in skeletal muscle (Fig. 5A), whereas levels of either nuclear or mitochondrial OGG1 were not significantly influenced by a HFD in liver (Fig. 5B). Regarding APE1 levels, similarly to increased of nuclear OGG1 in skeletal muscle, HFD also markedly increased levels of nuclear APE1 in both skeletal muscle and liver (Fig. 5 C and D). By contrast with mitochondrial OGG1, HFD significantly decreased mitochondrial APE1 in skeletal muscle (Fig. 5C), whereas the decrease in the mitochondrial APE1 level in liver was not statistically significant (Fig. 5D).

### HFD Animals have Increased ER Stress and Protein Degradation in Both Skeletal Muscle and Liver

**Figure 6:**
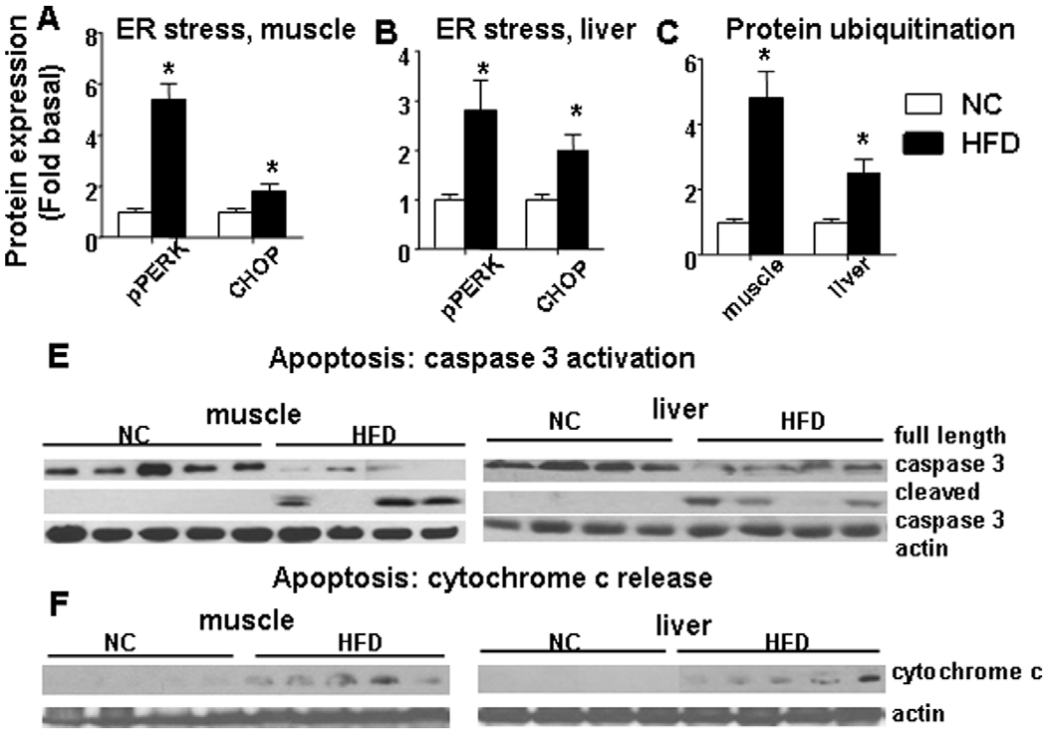
HFD induced markers of ER stress, protein degradation and increased apoptosis in skeletal muscle and liver. pPERK/PERK and CHOP levels were increased after a HFD in both skeletal muscle (A) and liver (B). (C) HFD increased protein ubiquitination (a marker of both ubiquitin-proteasome and autophagy-induced protein degradation) in both skeletal muscle and liver. The average results ± SE are shown. (* p<0.05 vs corresponding NC, n = 6–9 mice per group). (E) Caspase 3 and cleaved caspase 3 antibodies were used to recognize full length (35 kD) caspase 3 and cleaved caspase 3 large fragment (17 kD), respectively. (F) Western blot of cytochrome c release into cytosol. Cytosolic fractions isolated from skeletal muscle and liver from NC/HFD fed mice are shown. Equal loading was confirmed using anti actin antibody (n = 4–5 mice per group).

Since several studies have shown a structural communication between mitochondria and ER, and given the fate of dysfunctional mitochondria in autophagy [19], we hypothesized that the increased mitochondrial dysfunction and oxidative stress in HFD animals may be associated with ER stress, protein degradation and autophagy. Indeed, we found that a prolonged HFD induced activation both of ER stress, as shown by increased protein expression of CHOP and increased phosphorylation of PERK **(** Fig. 5A and B) and protein ubiquitination, which is a marker of both ubiquitin-proteasome-dependent and autophagic protein degradation [20]–[21] in both skeletal muscle and liver (Fig. 6C).

### HFD Leads to Increased Apoptosis and Loss of Proteins Involved in Insulin Signaling in Skeletal Muscle and Liver

**Figure 7:**
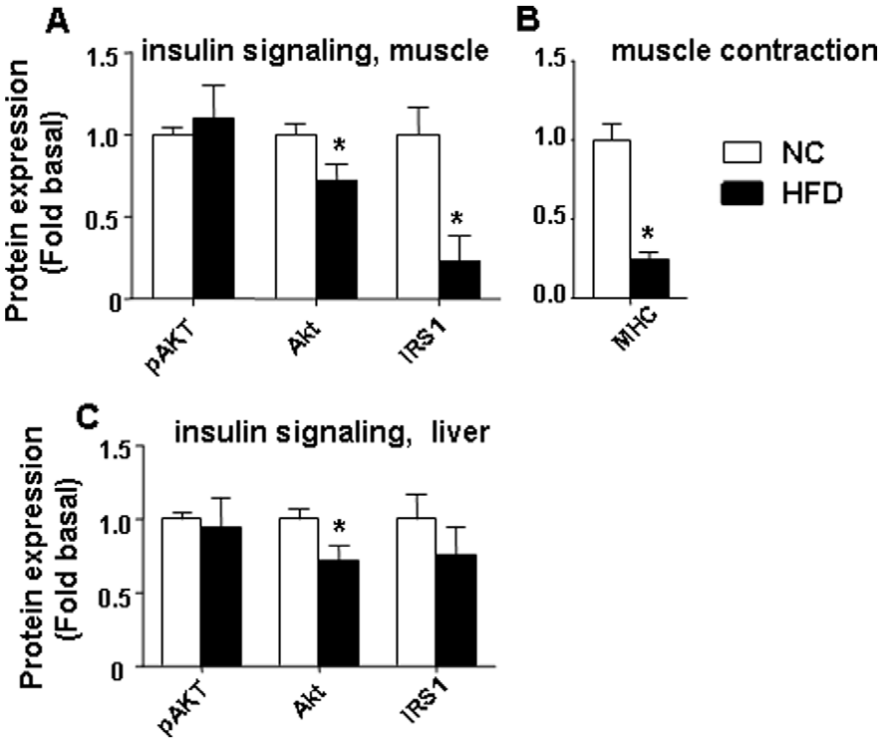
Effect of HFD on content of some proteins involved in insulin signaling in skeletal muscle and liver. (A) and (C) displays data for pAkt, Akt and IRS-1. (B) a HFD reduced MHC protein expression in skeletal muscle. The average results ± SE are shown. (*p<0.05 vs corresponding NC, n = 6–9 mice per group).

Previously, using cultured skeletal muscle L6 myotubes, we established the saturated FFA palmitate induced apoptosis *in vitro* [13], [22]–[23]. There is a discrepancy in the data evaluating HFD-induced apoptosis in skeletal muscle *in vivo*, with two reports indicating increased apoptosis in rodent skeletal muscle after a HFD [7]–[24] and one indicating an absence of apoptosis [25]. These discrepancies could be due to several reasons, including study design, type and duration of the diet, species, and muscle fiber differences. We believe that similarly to our study, presence of apoptosis correlates with the longer duration of HFD feeding (16 weeks) in the study by Bonnard [7] whereas 12 weeks of HFD [25] diet did not induce apoptosis in skeletal muscle. We have evaluated apoptosis in skeletal muscle using Western blot analysis of caspase 3 activation and cytochrome c release from mitochondria. Sixteen weeks of HFD increased apoptosis as shown by caspase 3 cleavage (Fig. 6E) and increase of cytochrome c in the cytosolic fractions isolated from both skeletal muscle and liver (Fig. 6F). In addition, we have shown that 16 weeks of HFD feeding significantly reduced the total content of two major proteins in the insulin signaling pathway, IRS-1 and Akt in skeletal muscle (Fig. 7A) and Akt in liver (Fig. 7C). Also, we found that basal pAkt level were unaffected by HFD diet in both skeletal muscle and liver (Fig. 7A and C). Moreover, HFD significantly decreased protein expression of myosin heavy chain (MHC) in skeletal muscle (Fig. 7B). We think that the reduced expression of MHC could lead to diminished contractile activity in skeletal muscle.

## Discussion

Excess lipid accumulation has been associated with mitochondrial dysfunction, oxidative stress and development of IR in skeletal muscle in obese rodent models and humans [6], [7]–[26]. Furthermore, recent studies on obese rodents have shown that mitochondrial dysfunction precedes IR and hepatic steatosis and contributes to the natural history of non-alcoholic fatty liver disease [8]. Although obesity, and consequently, its metabolic disorders, caused by overnutrition and sedentary life style, became an epidemic worldwide, the underlying mechanisms have yet to be elucidated. Since skeletal muscle and liver both play a crucial role in the development of IR [5], our study was designed to further clarify the molecular basis for the mechanisms responsible for the origin and pathways leading to the activation of IR in these organs. Our study makes several important new contributions to the field of skeletal muscle and liver IR. First, we provide evidence that a prolonged HFD decreased mtDNA copy number and increased mtDNA damage in skeletal muscle. Also, a HFD induced substantial mtDNA damage in liver samples, although the copy number of mtDNA was unaltered. The difference in effects of HFD on mtDNA copy between skeletal muscle and liver could be due to many reasons, including the fact that HFD induced more profound oxidative stress in skeletal muscle compared to liver. Despite extensive studies on mitochondrial function in both skeletal muscle and liver, until recently the quantitative aspects and integrity of mtDNA, and mtDNA repair mechanisms have received little attention in obesity and diabetes research. Although previous publications have shown a decrease in mtDNA content in skeletal muscle and liver models of DOI IR [7]–[8], all these reports should be interpreted with caution because they have not distinguished mtDNA damage from copy number. Therefore, the crucial question regarding whether a HFD caused mtDNA damage or reduced copy number had yet to be resolved until our report. In addition, there are contradictory data regarding mtDNA content in skeletal muscle from HFD fed animals [7], [11]–[27]. In agreement with our finding, the DIO study performed by Bonnard et al [7] has shown a significant reduction in mtDNA content in gastrocnemius muscle from IR obese mice, whereas another study has reported that a HFD caused IR despite an increase in muscle mtDNA, proteins and mitochondria [11]. These discrepancies between skeletal muscle mtDNA content could be due to several factors, including study design, type and duration of the diet, species, and muscle fiber differences. Consistent with this notion, a very recent study has demonstrated that the ratio of mtDNA to nDNA was unaltered in glycolytic skeletal muscle, while it was significantly reduced in oxidative skeletal muscle and liver from a genetic model of obesity, db/db mice [27]. Also decreased mtDNA content was found in the skeletal muscle from type 1 and type 2 diabetic patients [28].

Our study was designed to analyze not only the copy number of mtDNA but also the level of mtDNA and nDNA damage in both liver and skeletal muscle in animals fed a HFD. Even though a potential limitation of this study is that only 16 week endpoint variables were examined, we want to point out that our study was not designed to establish the cause-effect relationship between mitochondrial DNA damage, dysfunction and developing of IR in both liver and skeletal muscle. For this, additional studies are required which involve mouse genetics models of mitochondrial DNA repair enzymes, and evaluation of mitochondrial DNA damage and dysfunction at different time points on HFD prior to the occurred IR.

To the best of our knowledge, this is the first study to evaluate the effect of prolonged HFD on the efficiency of BER enzymes in both nuclear and mitochondrial fractions isolated from liver and muscle. First, we found that HFD significantly increased protein content for both mitochondrial and nuclear OGG1 in skeletal muscle. Our findings are in agreement with previous report indicated increase of mitochondrial OGG1 in humans with type 2 diabetes [29]. Second, HFD markedly upregulated levels of nuclear APE1 in both skeletal muscle and liver and decreased mitochondrial APE1 in skeletal muscle whereas the decrease in the mitochondrial APE1 level in liver was not statistically significant. Our data clearly indicate existence of different mechanisms for regulating BER machinery in nucleus and mitochondria in skeletal muscle and liver.

Consistent with previous reports [6]–[7], we have shown that a HFD induced dysfunctional mitochondria, as shown by the decline in ATP levels and reduced content for key mitochondrial proteins, including antioxidants and porin. Our findings also are consistent with a very recent study which reported a decline in mtDNA and mitochondrial dysfunction in oxidative skeletal muscle in a mouse genetic model of obesity, db/db mice [27]. Oxidative stress has been shown to be an initiator and major contributor to both ER stress [30]–[31] and autophagy [19], although the mechanisms that promote the activation of these signaling routes and upstream targets are not completely defined. Increased ROS are considered to act as local messengers between ER stress and mitochondria [32]. Activation of ER stress has been shown in liver of leptin-deficient ob/ob mice [33]. Regarding skeletal muscle, there are conflicting data as to whether a HFD diet induced ER stress, which possibly can be explained by the difference in study duration and diet composition [17]–[33]. It is widely accepted that ER stress induces mitochondrial dysfunction [32]–[34]. Furthermore, it has been shown that mitochondrial dysfunction increased the level of ER stress markers in adipocytes [20]. The ubiquitin-proteasome and autophagy-lysosome systems are two major protein degradation pathways. During the degradation of misfolded proteins, the ER is connected to both the ubquitin-proteasome and to autophagy [30]. Since we have shown increased oxidative stress and mitochondrial dysfunction in liver and muscle tissues after a HFD, it is tempting to speculate that markers of ER stress and protein degradation also are activated; therefore, our next studies were designed to clarify this issue. Consistent with previous data [17], we have shown increased phosphorylation of PERK (in both liver and gastrocnemius muscle), an ER stress sensor which initiates the unfolded protein response also termed as ER stress. Also, a HFD induced activation of JNK, which is considered to be a marker of both increased oxidative and ER stress [38]. In addition, we have shown that a HFD increased ubiquitination of proteins, which is considered to be a marker of both ubiquitin-proteasome and autophagic degradation of proteins [20]–[21].

Although our study was not designed for a detailed comparative and correlative analysis, we would point out that activation of markers of ER stress and protein degradation was greater in muscle, which we believe correlates with a more profound oxidative stress in muscle induced by a HFD. In addition to the increase of ubiquitination of proteins, we found that apoptosis also was increased in both skeletal muscle and liver, which agrees with previous reports in other obese rodent models which documented that a HFD induced apoptosis in skeletal muscle [7]–[24] and liver [35]. Consistent with increased markers for protein degradation, total protein levels for Akt and IRS-1, two major proteins of the insulin signaling pathway, were decreased after a HFD, possibly as result of the increased protein degradation which likely would contribute to the impaired insulin signaling in the muscle after HFD. Interestingly, the basal pAkt level was not significantly influenced by HFD feeding in both skeletal muscle and liver. In addition, MHC, one of the markers for both differentiated skeletal muscle and for skeletal muscle contractile function, was significantly reduced following a HFD, also suggesting there is an increase in muscle loss.

In conclusion, this study provides new insight into the mechanisms required for development of IR at two major sites, skeletal muscle and liver. Our results further support the hypothesis that HFD induced mitochondrial dysfunction and oxidative stress in both liver and skeletal muscle and that new therapies are imperative to protect mitochondria and, thus, decrease the development of IR.

## Supporting Information

Figure S1 **HFD mice have impaired glucose tolerance and are insulin resistant.** (A) oral glucose tolerance (OGTT) and (B) insulin tolerance tests (ITT). The top part of each panel indicates the corresponding Area Under the Curve (AUC) for the performed analyses. Data represent the means ± SE. If the error bar is not visible, the standard error is smaller than the symbol. * p<0.05 vs NC. (n = 12–15 mice per group).
(TIF)

Figure S2 **No detectable contamination was observed in the nuclear, cytosolic and mitochondrial fractions isolated from liver and skeletal muscle.** Nuclear, mitochondrial and cytosolic fractions were isolated from skeletal muscle and liver of NC/HFD fed mice and analyzed by Western blot for the purity of fractions using indicated antibodies.
(TIF)

Table S1 Characteristics of the mice. Data are expressed as means ± SE. (* p<0.05 ***vs*** **NC, n = 10–15 mice per group).**
(DOC)
