# Supplementary material for: Mitochondrial DNA Damage and Dysfunction, and Oxidative Stress Are Associated with Endoplasmic Reticulum Stress, Protein Degradation and Apoptosis in High Fat Diet-Induced Insulin Resistance Mice
Source: PLoS One. 2013 Jan 16;8(1):e54059. doi: 10.1371/journal.pone.0054059 (PMC3546973; doi:10.1371/journal.pone.0054059)
Supplement: Table S1 — Characteristics of the mice. Data are expressed as means ± SE. (* p<0.05 vs NC, n = 10–15 mice per group). (DOC) [file pone.0054059.s003.doc]

**Table S1.**

| **Characteristic** | **NC** | **HFD** |
| --- | --- | --- |

Body weight (g) 27.7 + 0.44 38.07 + 0.73*

Glucose (mg/dl) 189.5 + 7.7 267.8 + 9.82*

Insulin (ng/ml) 0.7 + 0.06 1.518 + 0.27*

Triglycerides (mg/dl) 34.32 + 2.33 80.2 + 5.4*

FFA (mM) 0.51 + 0.06 1.08 + 0.1*
